# Editorial: Glial heterogeneity: impact on neuronal function and dysfunction

**DOI:** 10.3389/fnana.2023.1249919

**Published:** 2023-07-13

**Authors:** Paola Bezzi, Valerio Magnaghi, Rosa Chiara Paolicelli, Jean-Pierre Hornung

**Affiliations:** ^1^Department of Fundamental Neurosciences, FBM, University of Lausanne, Lausanne, Switzerland; ^2^Department of Pharmacological and Biomolecular Sciences, Universita degli Studi di Milano, Milano, Italy; ^3^Department of Biomedical Sciences, FBM, University of Lausanne, Lausanne, Switzerland

**Keywords:** astrocytes, oligodendrocytes, microglia, schwann cells, mitochondria and metabolism, development, synaptic modulation, satellite cells

In addition to their contribution to neuronal homeostasis, the implication of central nervous system (CNS) non-neuronal cells in the building, regulation and maintenance of the neuronal network of the brain has been widely investigated in the recent years, involving all types of glial cells. They contribute during development to neuronal migration, axonal guidance, synaptic pruning and in the mature CNS, they are involved in synaptic modulation, adjustment of axonal conductance, among factors modulating the appropriate neuronal activity to the local circuitry and the connectivity within specific networks. Dysfunction of the cellular properties of glial cells contributes to pathologies impacting cognitive and sensorimotor functions. The regional heterogeneity among glial cells contributes also to specific properties of the neuronal networks in the various parts of the brain.

This series includes seven original research and review articles covering different aspect of the topic outlined above.

The review by Shafqat et al. provides a comprehensive overview on the dual roles of the glial scar in the context of spinal cord injury (SCI). Although the glial scar formation is commonly regarded as a mechanism to protect the nervous system after injury, it has been for long time considered a process hindering axonal regeneration. In this work, new evidence is discussed for vital neuroprotective functions of glial and stromal responses to SCI. Glial heterogeneity and beneficial outcome of reactivity in scar formation are extensively examined, with a special focus on astrocytes, microglia and oligodendrocytes. Finally, this review proposes and discusses targeted manipulations of cellular components of the glial scar to enhance axonal regeneration and improve SCI outcomes.

The paper by Elsayed et al. deals with the mechanisms at the base of the diabetes-induced central neuropathy in the spinal cord and the pharmacological use of curcumin for its innovative treatment. Diabetes is a multifactorial disease with high prevalence in the global population, entailing a classic peripheral and central neuropathy. The identification of new medical approaches, therefore, is a priority to prevent and, hopefully, tackle microglial/glial activation as well as neuronal degeneration induced by diabetes in the spinal cord. Curcumin, which is a novel and promising natural compound, proved effective in counteracting the spinal neuropathy, via suppression of the neuroinflammatory NF-kB signaling, as well as suppression of gliosis and neuronal apoptosis.

The paper by Thapaliya et al. developed a detailed biophysical model replicating key observations about Na+ and Ca^2+^ homeostasis in cortical and hippocampal astrocytes with the aim of providing deeper insight into the role of NMDA and AMPA receptors in the observed heterogeneity of Na+ and Ca2+ homeostasis in astrocytes. They also deciphered the effects of heterogenous expression of NMDA receptors on Na+ and Ca2+ dynamics and ATP consumption in the cortical and hippocampal astrocytes when stimulated by a high concentration of glutamate or NMDA (with Na+/K+-ATPase suppressed) as would be the case in numerous pathologies such as ischemic stroke, migraine, and traumatic brain injury.

The mini review by Andreeva et al. describes the different types of satellite glial cells (SGS) found in sensory and autonomic ganglia, reflecting the variety of neurons they surround and their involvement in pain conditions. Hyperexcitability and low threshold stimulation level of sensory neurons affected by nerve injury is associated changes in uptake by SGC of glutamate and various ions. The increased release of ATP and proinflammatory cytokines is associated with pain transmission.

The review by Tang et al. reports on the involvement of astrocytes in the pathogenesis of glaucoma. Although intraocular pressure (IOP) and aging are major risk factors, some patients still develop glaucoma after lowering IOP. Heterogenous populations of astrocytes display up- or down-regulations of molecular changes over time and space which impact retinal ganglionic cell survival in glaucoma. Several second messenger pathways have either beneficial (i.e. STAT3, complement C3) or detrimental (i.e. caspase 8, NF-RB, TNFα) opening up strategies for protective treatment for glaucoma patients in the future.

The paper by Plastini et al. tested the hypothesis that intrinsic dysfunction in oligodendrocytes (OLs), may contribute to etiopathology of the primary progressive multiple sclerosis (PPMS), in mature OLs obtained from differentiated human induced pluripotent stem cell (hiPSC) lines derived from PPMS and healthy individuals. The authors found that PPMS derived OLs displayed hundreds of differentially expressed genes compared to control OLs, including the inflammasome component Nlrp2 thus suggesting that mature OLs in PPMS carry intrinsic abnormalities that could contribute, at least in part, to the pathophysiology of this form of the disease.

In the research article by Decoeur et al., the effects of n-3 polyunsaturated fatty acids (n-3 PUFAs) on microglia in the developing white matter are discussed. Since low maternal intake of n-3 PUFAs has been associated with deficits in brain myelination, the authors set to investigate whether microglia, which notably contribute to this process, are implicated. Combining light and electron microscopy, this work shows that n-3 PUFAs maternal deficiency reduces microglial density and interaction with myelin in the *corpus callosum* of the offspring. In addition, mitochondrial content per microglia is increased in association with n-3 PUFAs deficiency. Taken together, these findings indicate that maternal n-3 PUFA deficiency affects white matter microglia in the early post-natal brain, highlighting the critical role of maternal diet for microglial modulation and offspring brain development.

This series of articles illustrates how the diversity in glial cells contributes to normal function or dysfunction at different stages of development and different brain regions and systems. It illustrates how much non-neuronal cells contribute, with their diversities and distributions, to the building, maintenance, and functional regulation of the neuronal circuits.

## Author contributions

All authors listed have made a substantial, direct, and intellectual contribution to the work and approved it for publication.

